# Insights into Object Semantics: Leveraging Transformer Networks for Advanced Image Captioning

**DOI:** 10.3390/s24061796

**Published:** 2024-03-11

**Authors:** Deema Abdal Hafeth, Stefanos Kollias

**Affiliations:** 1 School of Computer Science, University of Lincoln, Lincoln LN6 7TS, UK; dabdalhafeth@lincoln.ac.uk; 2 School of Electrical & Computer Engineering, National Technical University of Athens, 15780 Athens, Greece

**Keywords:** image captioning, deep learning, transformers, attention, vision language

## Abstract

Image captioning is a technique used to generate descriptive captions for images. Typically, it involves employing a Convolutional Neural Network (CNN) as the encoder to extract visual features, and a decoder model, often based on Recurrent Neural Networks (RNNs), to generate the captions. Recently, the encoder–decoder architecture has witnessed the widespread adoption of the self-attention mechanism. However, this approach faces certain challenges that require further research. One such challenge is that the extracted visual features do not fully exploit the available image information, primarily due to the absence of semantic concepts. This limitation restricts the ability to fully comprehend the content depicted in the image. To address this issue, we present a new image-Transformer-based model boosted with image object semantic representation. Our model incorporates semantic representation in encoder attention, enhancing visual features by integrating instance-level concepts. Additionally, we employ Transformer as the decoder in the language generation module. By doing so, we achieve improved performance in generating accurate and diverse captions. We evaluated the performance of our model on the MS-COCO and novel MACE datasets. The results illustrate that our model aligns with state-of-the-art approaches in terms of caption generation.

## 1. Introduction

Image captioning models aim to automatically describe the visual content within a provided image with coherent and accurate textual descriptions. This task represents a standard example of multi-modal learning, bridging the domains of Computer Vision (CV) and Natural Language Processing (NLP). Image captioning models have utility across diverse domains, with application including assistance to individuals with visual impairments [1,2], automatic medical image captioning [3] and diagnosis [4], and enhancing human–computer interactions [5]. Motivated by the achievements of deep learning techniques in machine translation [6], the majority of image captioning models adopt the encoder–decoder framework coupled with a visual attention mechanism [7,8]. The encoder transforms input images into fixed-length vector features, while the decoder decodes image features into descriptions, progressing word by word [9,10,11,12,13].

In the past few years, researchers have adopted a pre-trained Convolutional Neural Network (CNN) as an encoder for extracting high-level features from the input image, with a Recurrent Neural Network (RNN) serving as the decoder [9,10]. Initially, Anderson et al. [11] introduced the use of the Faster R-CNN object detector for extracting features at the regional level. Due to its substantial advantages, this approach became widely adopted in subsequent works. However, there are still shortcomings regarding regional-level features and the encoder of the object detector. Regional-level features may not capture specific and subtle elements that contribute to a more comprehensive understanding of the image content [14]. Additionally, the encoder treats the image as sequences of visual features and does not preserve the spatial semantic information of the image. This can result in inaccurate or ambiguous captions, especially when objects in the image have spatial semantic relationships, as noted by Anderson et al. [11,15].

Recently, the main approach in image captioning models has been the use of Long Short-Term Memory (LSTM) [16] decoders with a soft attention mechanism [10]. However, drawbacks related to the training efficiency for handling long-term dependencies and inherited issues associated with sequential processing of LSTMs constrain the effectiveness of such models. Motivated by the achievements observed with the multihead self-attention mechanism and the Transformer architecture [17] in Natural Language Processing (NLP) tasks, numerous researchers have started integrating multihead self-attention into the LSTM decoder [12,13] or directly employing the Transformer architecture as the decoder [14,18,19] in image captioning models.

Especially, Transformer architecture gradually shows extraordinary potential in CV tasks and multi-modal tasks [14,20,21,22]. Researchers have proposed various methods that provide a new choice for encoding images into vectors of features. Nevertheless, they neglect image content semantic information in encoder Transformer modules and focus only on image visual features extracted by CNN and object detectors. Acknowledging the constraints associated with semantic image representation, we employ a Transformer-based image captioning model and incorporate external semantic knowledge representation for image objects in the encoder Transformer module. This is aimed at capturing meaningful relationships between image objects and subsequently improving the caption generation process. In encoder, we adopt faster R-CNN as an image object detector to extract objects’ visual features within the image and the class label of these detected objects. Then, we generate object semantic word embedding representation similar to [15] from the class label by using an external knowledge base. Both of these objects, visual features and object semantic word embedding representation, serve as input to the encoder Transformer module, allowing it to focus attention on relevant regions when generating image captions. In contrast to [15], in decoder, we directly adopt a Transformer decoder in machine translation [17] to generate captions. This captioning model design enhances the performance of image captioning by enabling parallel processing of information. This parallel approach is more efficient for sequence-to-sequence tasks compared to LSTM models. Also, it empowers the model to make more informed and contextually relevant decisions when generating descriptive text for the image content by combining the encoder’s context vector with the encoding representation of the current word, resulting in the output text [20,23].

We validate our model via the MS-COCO [24] offline “Karpathy” test split, which demonstrates the effectiveness of our proposed model. Also, we use a private novel MACE [25] dataset for model generalization application. A comprehensive set of experiments, as well as quantitative and qualitative analyses, provide insights into the effectiveness of semantic attention image captioning models in visual captioning tasks.

Our main contributions are summarized as follows:

- We create a Transformer-based image captioning model that integrates the external semantic knowledge representation of image objects into the encoder Transformer. This incorporation enhances the encoder and decoder Transformers’ capability to focus their attention on relevant regions and capture the meaningful relationships between image objects throughout the image captioning generation process.
- We conduct a linguistic social word analysis for the generated captions, offering valuable insights into the efficacy of using the proposed model in vision and language tasks.
- We extend the applicability of the proposed model and generate a description for the MACE visual captioning dataset. This newly archival dataset contains significant historical videos and scenes.

The remainder of this paper is organized as follows: Section 2 presents background and related work. Section 3 describes the model architecture. This is followed by the experiments and results in Section 4. Section 5 provides a discussion on the achieved outcomes. Model generalization is presented in Section 6. The paper’s conclusions and future work ideas are provided in Section 7.

## 2. Background and Related Works

In the past few years, motivated by the achievements of encoder–decoder frameworks in machine translation [6], a diverse range of approaches adopting the encoder–decoder model in image captioning have emerged, achieving significant success. The conventional encoder–decoder models [9,26] employ a CNN as the encoder and an LSTM as the decoder, incorporating sequence-to-sequence connections. Subsequently, there have been numerous efforts aimed at advancing the encoder–decoder paradigm. Anderson et al. [11] introduced a bottom-up mechanism for encoding with LSTM for decoding, facilitating attention calculation at the visual object level rather than initially across a uniform grid of CNN features [10,27]. Moreover, Zhang et al. [28] introduced a visual relationship attention mechanism employing contextualized embeddings for visual objects. In the decoding phase, Xu et al. [10] utilized LSTM to decode the convolutional features of an image, employing both hard and soft attention mechanisms to effectively highlight crucial regions. Lu et al. [27] proposed incorporating a visual sentinel into the encoder–decoder framework for automatically regulating adaptive attention. Zhong et al. [29] suggested employing adaptive spatial information attention (ASIA) to improve the utilization of feature information within images by enhancing LSTM’s ability to grasp the spatial details of significant objects or entire images from both global and local viewpoints.

In addition to utilizing visual features, techniques that leverage semantic information have been shown to significantly enhance caption accuracy. These additional semantic data can originate from either the entire image [30,31] or specific visual elements within the image [11,32]. To maximize the utilization of object semantic details, Yao et al. [33] introduced Long Short-Term Memory with Attributes (LSTM-A), which incorporates attributes and visual features as inputs to LSTM, thus merging attributes into the effective CNN plus LSTM image captioning framework. Li et al. [34] proposed a visual–semantic LSTM model that incorporates an attention mechanism to focus on visual semantic information. Furthermore, certain methods employing Graph Convolutional Networks (GCN) introduce semantic object relationships into the encoder–decoder architecture, enhancing semantic information utilization. Yao et al. [35] suggested using GCN to incorporate semantic and spatial object relationships into the encoder. For a different approach to integrating semantic information, Hafeth et al. [15] proposed involving external semantic knowledge bases representation for image objects’ labels to enrich visual attention in image encoders. Yang et al. [36] introduced the Scene Graph Auto-Encoder (SGAE), which leverages semantic information to construct a dictionary, providing essential linguistic knowledge to guide the encoder–decoder process. Alternatively, instead of combining integrated semantic and visual information, Guo et al. [37] proposed Visual Semantic Units Alignment (VSUA) to fully exploit alignment between word embeddings and integrated visual semantic units for image captioning.

Traditional encoder–decoder frameworks, characterized by recursive dependencies, encounter challenges in parallelization during training, resulting in diminished algorithmic efficiency. Consequently, the Transformer model [17], which naturally accommodates the encoder–decoder paradigm and supports parallel training, emerged as a solution for image captioning tasks. Sharma et al. [38] suggested the integration of the Transformer model into image captioning, demonstrating its efficacy. Additionally, the Transformer leverages spatial relationships extensively to enhance captioning accuracy. Herdade et al. [39] proposed an object relation Transformer that explicitly incorporates spatial relationships among detected objects using geometric attention in the encoder phase. He et al. [8] introduced a model based on image-Transformer encoder, aiming to enhance multihead attention by considering other relative spatial graph Transformer layers among image regions using only region visual features as input. Huang et al. [12] proposed AoANet, introducing an additional attention mechanism by employing gating on the information, thereby enhancing the model’s ability to focus on relevant information. For a different approach to encoder attention, Cornia et al. [18] utilized attention mechanisms to integrate outputs from multiple encoder layers. To maximize semantic information utilization in the Transformer, Li et al. [40] introduced EnTangled Attention (ETA), enabling simultaneous exploitation of semantic and visual information in the decoder. Zhang et al. [20] introduced the Multi-Feature Fusion-enhanced Transformer, a new approach to image captioning. Their model aims to boost Transformer performance in both encoder and decoder stages. By incorporating multi-feature fusion mechanisms, the model aligns specific visual and semantic features while also improving word organization. These enhancements contribute to more detailed and accurate descriptions. Luo et al. [23] introduced the SCD-Net model, which enhances the synchronization of visual content and text across three stacked Transformers: a visual encoder, a semantic Transformer, and a sentence decoder. Their objective is to produce captions that are both coherent and semantically rich.

Based on the above reviews, it is apparent that few methods of techniques fully leverage the image semantic representation within Transformer-based image captioning methods. In addition, the Transformer architecture in Natural Language Processing demonstrates the ability to capture complex semantic connections. Inspired by this observation, we propose a new Transformer-based model specifically designed for image captioning. The proposed model employs a Transformer network for both encoder and decoder architecture, and integrates a semantic encoder Transformer to enhance semantic understanding to generate detailed captioning output.

## 3. Model Architecture

In this section, we provide the background information on the Transformer model, which serves as the foundation for our work (Section 3.1). Subsequently, we present an illustration of the used semantic knowledge graph (Section 3.2). Lastly, we explain the comprehensive architecture of our proposed model in detail (Section 3.3).

### 3.1. Transformer Model for Image Captioning

**Figure 1:**
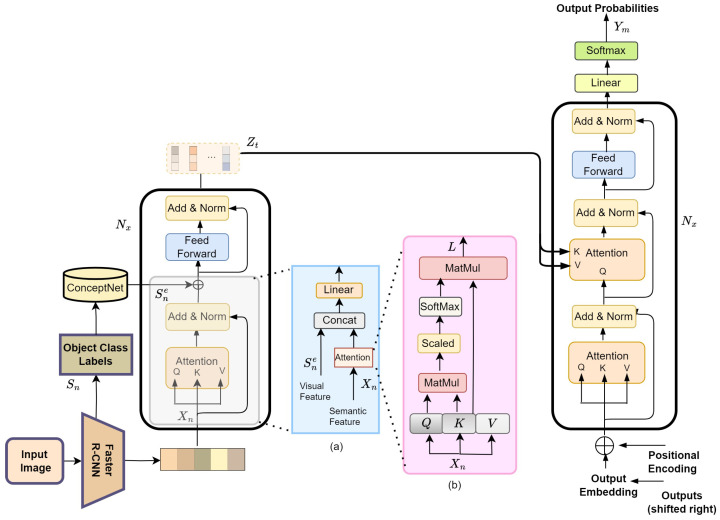
Overview of our proposed model. We first extract visual features $X_{n}$ and class labels $S_{n}$ of image objects utilizing Faster R-CNN [32]. Following this, we generate semantic representations of object class $S_{n}^{e}$ by leveraging the ConceptNet knowledge base [41]. Both representations are input into the Transformer encoder and then sequentially passed $Z_{t}$ to the Transformer decoder to generate the description word by word. Both sub-figures (**a**,**b**) show an attention module and a semantic attention module, respectively.

We employ the Transformer model for image captioning, comprising an encoder and a decoder (Figure 1). The encoder maps the input image representation $x=(x_{1},\ldots ,x_{n})$ to a sequence of continuous representations $z=(z_{1},\ldots ,z_{t})$. The decoder generates the output sequence $y=(y_{1},\ldots ,y_{m})$ for *z*. *x* represents the image visual features extracted from the input image, and *n* denotes the number of features. The features we utilized are known as bottom-up features, derived from the bottom-up attention model introduced by Anderson et al. [11]. *z* represents the output vector of the Transformer encoder, with a dimension of *t*. *y* corresponds to the output sentence generated by the Transformer decoder, with a length of *m*. Unlike other image captioning models, the Transformer model employs stacked self-attention and point-wise fully connected layers instead of recurrent layers for both the encoder and decoder. The Transformer model specifically employed in this paper is based on [17]. Additionally, the model’s input is replaced with features extracted from images.

Generally, the Transformer employs scaled dot-product attention to focus on relevant parts of the input sequence when generating the output, providing a way to capture dependencies and relationships within the data. This involves calculating the dot product between the query and key vectors, scaling it, applying a softmax to obtain attention scores, and then using these scores to weigh the corresponding values for each element in the input sequence [42]; the computational procedure can be illustrated as follows:

(1) $$Attention\left(Q,K,V\right)=softmax\left(\frac{QK^{T}}{\sqrt{d_{K}}}\right)V$$

In the given context, the attention inputs comprise the queries matrix *Q*, keys matrix *K*, and values matrix *V*, all derived from the input sequence. The respective dimensions of these matrices are $d_{Q}$, $d_{K}$, and $d_{v}$. To minimize the impact of the substantial value of $d_{k}$, a normalization factor of $\frac{1}{\sqrt{d_{K}}}$ is employed to push the softmax function into regions with small gradients. In practice, dot-product attention proves to be faster and more space-efficient due to its ability to be implemented through parallel optimization [17].

Furthermore, multihead attention is constructed based on the foundation of scaled dot-product attention [42]. It has the ability to acquire diverse representation subspaces at various positions. It consists of *h* identical attention heads, where each head functions as a scaled dot-product attention, independently applying the attention mechanism to queries, keys, and values. Subsequently, the outputs from the *h* attention heads are concatenated and then projected back to the original dimension, resulting in the ultimate values (Equations (2) and (3)).

(2) $$MultiHead\left(Q,K,V\right)=Concat\left(head_{1},\ldots ,head_{h}\right)W^{O}$$

(3) $$head_{i}=Attention\left(QW_{i}^{Q},KW_{i}^{K},VW_{i}^{V}\right)$$

where $W^{O}\in R^{hd_{v}\times d_{model}}$, $W_{i}^{Q}\in R^{d_{k}\times d_{model}}$, $W_{i}^{K}\in R^{d_{k}\times d_{model}}$, $W_{i}^{V}\in R^{d_{v}\times d_{model}}$ are projection matrices that can be trained. In order to minimize overall computational expenses, the approach outlined in [17] involves projecting the initial dimension of $d_{model}=512$ onto $d_{k}=d_{v}=d_{model}/h=64$, where *h* is set to 8.

The feed-forward network serves as another fundamental component, comprising a two-layer fully connected network featuring a ReLU activation function. This activation function is employed to enhance the network’s nonlinear capabilities [42], as specified in Equation (4), where $x^{out}$ is the output of a previous sub-layer.

(4) $$FFN\left(x\right)=FC(Dropout\left(ReLU\left(FC\left(x^{out}\right)\right)\right))$$

The encoder consists of N identical layers, each containing two sub-layers. The first sub-layer is a multihead self-attention mechanism, while the second sub-layer is a fully connected feed-forward network. Both sub-layers are accompanied by a residual connection [43] and a normalization layer. The residual connection improves the flow of information and gradients, enabling more effective training, preserving important features and better overall performance of the Transformer model.

The decoder, like the encoder, consists of a stack of *N* identical layers. Each decoder layer contains three sub-layers. In addition to the two sub-layers found in the encoder, the decoder introduces a third sub-layer that performs multihead attention over the encoder stack’s output. Similar to the encoder, residual connections followed by normalization layers are applied around these sub-layers. The masked multihead attention sub-layer ensures that predictions for position *i* rely solely on the known outputs preceding position *i*, achieved through a mask operation. This is because, during training, the Transformer generates words at position *i* using the ground truth words, whereas, during testing, it generates the word at position *i* based on the previously generated words. It is depicted in Figure 1.

To apply the Transformer model to the image captioning tasks, we take the pre-trained bottom-up attention features [11] as the representation of the input image. These visual features are extracted from an image using the bottom-up attention model to identify salient objects or regions within an image.

### 3.2. Leveraging Knowledge Graphs

The encoder Transformer model traditionally relies on visual embedding vectors as input. Typically, these visual embedding vectors, associated with individual objects in an image, are derived exclusively from the objects themselves, utilizing only their basic information.

In our work, we adopt an attention Transformer architecture comprising 6 blocks, as outlined by Vaswani et al. in [17], to more effectively encode input images. As proposed by Hafeth et al. [15], the attention mechanism is enriched by external semantic knowledge bases (KBs), such as ConceptNet5 [41], which provide semantic object word representations.

The integration of KBs offers access to a wealth of semantic knowledge, resulting in enhanced caption quality and accuracy. This integration allows for the visual and semantic features extracted from the visual inputs to be mapped into a common space, facilitating meaningful comparisons and combinations. In essence, supplementing the visual content with additional semantic knowledge and context leads to the generation of more coherent and meaningful captions.

To achieve this, we extract ConceptNet word embeddings by harnessing a ConceptNet knowledge graph [41]. These word embeddings encapsulate comprehensive information about the meanings and relationships of words in a compact vector format. Each word or concept in ConceptNet is assigned a high-dimensional vector representation, with similar words having closely positioned vectors, signifying their semantic similarity. These word embeddings capture various aspects of word meanings, encompassing synonyms, antonyms, hypernyms, and hyponyms. For instance, the vectors representing “dog” and “cat” are positioned closer to each other than those representing “dog” and “car”, reflecting the greater semantic similarities between dogs and cats. This approach allows us to incorporate not only the information of the object itself but also the information of its neighboring objects.

### 3.3. Transformer with Semantic-Based Model for Image Captioning

The architecture of the proposed image captioning model is illustrated in Figure 1 and outlined in Algorithm 1. Training dataset has two types of input modalities, input image and caption(s) for that image only. We explain the process to extract semantic features representing image objects in the remaining part of the section.
**Algorithm 1:** Caption Generating Procedure
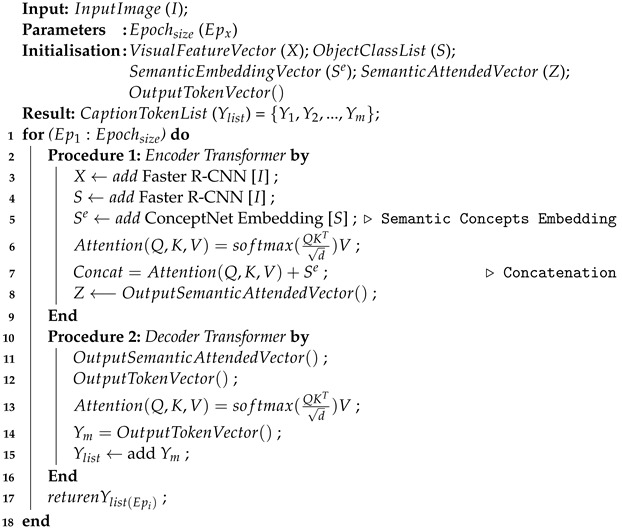


The proposed model has a dual stream of encoder to encode visual information and a single stream of decoder to decode the input image caption. The encoder uses a popular object detection architecture, the Region-based Convolutional Neural Networks Faster R-CNN model. This utilizes a deep Residual Network (ResNet) [43] as a convolutional backbone network to extract both visual feature map and class label for detected object in input image. The object detector network has been pre-trained on both the Imagenet dataset [44] and Visual Genome dataset [45]. The combination of Faster R-CNN and ResNet has demonstrated outstanding performance in object detection tasks, achieving state-of-the-art results [11].

Given an input image *I*, Faster R-CNN extracts features for the detected objects *N*, where $R^{N\times 2048}$ is extracted object features vector. These visual features are represented as one stream of encoder and used as part of the input sequence for the attention-based Transformer. They are projected to $P^{N\times 512}$ using a feed-forward layer and followed by a stack of six Transformer layers. Each layer consists of a self-attention layer and a feed-forward layer with residual connections and layer normalization, as explained in Section 3.1. Consequently, the visual attended vector $L^{N\times 512}$ is the output for each individual Transformer attention layer.

In the other stream, for input image *I*, Faster R-CNN predicts class label for detected objects $S_{n}$ as a list of words. These are transformed as word embedding vectors $S_{n}^{e}$ by using ConceptNet embedding [41]. These word embedding vectors are depicted as dense numerical vectors in a continuous 300 multi-dimensional space $R^{S_{n}^{e}}\times 300$. The resulting word embeddings encode information about the meaning and relationships of the objects’ semantic words in a dense vector format, as explained in Section 3.2.

To enhance Transformer layer, a fusion strategy is devised to integrate the two representations of input image, visual attended representation and semantic word representation. For the channel connect strategy, these two feature representations are concatenated and then reduced to the model hidden dimension with a linear matrix, as shown in Figure 1.

By fusing these modalities, we can leverage the complementary information they provide about the image content. Visual features offer fine-grained details about the visual content, while semantic word representations provide higher-level understanding and contextual information. As a consequence, this leads to the generation of captions that are more accurate, contextually relevant, and enriched with both visual and semantic details.

## 4. Experiments and Results

### 4.1. Datasets

Microsoft-COCO Data: We evaluated our method on the most popular image captioning benchmark, the Microsoft Common Objects in Context (MS-COCO) 2014 dataset [24]. Most recent works prefer to experiment on the MS-COCO dataset [46] due to its substantial size compared to other datasets like Flickr8k [47] and Flickr30k [48].

For offline performance comparisons, we followed the ‘Karpathy’ COCO data split [49], where 82,783 images were used for training, 5000 images for validation, and 5000 images for testing. This particular dataset split plays a crucial role in assessing the performance of image captioning models. It has become a standard in the field for evaluating various methodologies in image captioning. Each image corresponded to five manually annotated captions. Pre-processing of the textual data involved converting image captions to lowercase, sentence tokenization, punctuation removal, and the elimination of words occurring fewer than five times in total.

MACE Data: To address the lack of research on generating captions for historical visual data and to facilitate the search and exploration of historical multimedia collections, we hypothesized that our proposed semantic attention model can automatically describe historical visual content. To evaluate this hypothesis, our study introduced the Media Archives for Centre England (MACE) dataset [25]. This dataset comprises historical films, each associated with title classifications and text descriptions. Human annotators were tasked to provide one or more independent descriptions for each video, aiming to describe the objects and events in the scenes through (a) sentence(s).

While the MACE corpus was not initially designed for video or image captioning, our aim was to utilize it for training and evaluating our proposed image captioning generation model. We successfully converted video data into individual frames and generated frame–text pairs, which serve as input for the image captioning model. This conversion involves breaking down a video file into individual image frames. About 20 frames per second was selected as the frame rate. They are stored as individual image files in formats like PNG. A multimedia library like OpenCV in Python 3 is used for this purpose. Then, for each frame, one or more captions are selected from the previously provided video descriptions. This process involves creating an individual scene description by combining information from previous or later frame descriptions. We ensured that each caption satisfied the requirements of describing the events and objects in the image and was true for the given image. In total, 12,492 frame–text pairs were extracted for 25 videos. On average, each film contained approximately 499.7 frames. The resulting data were split into 1249 images for validation, another 1249 images for testing, and the remaining 9994 were used for training. The dataset can be provided by request to the authors, with the permission of MACE.

### 4.2. Evaluation Metrics

More recent approaches employ evaluation metrics that perform well in image captioning tasks. They assess the quality of produced captions by comparing them with reference captions. To evaluate the performance of the proposed model and validate our results, we used metrics such as BLUE [50], which is denoted as B@N (N = 1, 2, 3, 4), ROUGEL [51], CIDEr [52], and METEOR [53].

In consequence, these evaluation metrics play pivotal roles in assessing the efficacy of generated captions and in various Natural Language Processing tasks [54]. Higher scores indicate better alignment between candidate and reference captions. BLEU, with its emphasis on the alignment of n-grams, serves as a valuable tool in machine translation, providing a measure of how well the generated text aligns with reference captions for different n-gram orders (from 1 to 4). METEOR incorporates precision, recall, stemming, and synonymy to assess the quality of generated captions. It provides a balanced measure of fluency and relevance. Originating from the domain of text summarization, ROUGE takes a comprehensive approach, evaluating the concurrence of n-grams, word sequences, and word overlap to offer a holistic perspective on content similarity in both generated and reference captions. CIDEr extends beyond conventional n-gram assessments by incorporating semantic similarity, utilizing a weighted term frequency-inverse document frequency (TF-IDF) approach for assessing the effectiveness of the generated caption in capturing a diverse array of linguistic expressions.

### 4.3. Experiment Details

We conducted multiple experiments to assess the accuracy of the generated captions framework using standard language evaluation metrics for visual captioning techniques. The baseline framework for the proposed model consists of a Faster-RCNN with a ResNet-101 object detector, combined with an encoder semantic Transformer and decoder Transformer. Our image encoder and caption decoder stacked six layers with eight attention heads. The hidden unit dimension of multihead attention was 512. We trained our model for 50 epochs for all the experiments on the MS-COCO test split and MACE test set.

#### 4.3.1. Quantitative Evaluation

This section represents the performance of the employed method on MS-COCO offline evaluation. We compared the current findings to the following prior research results. The sequence-to-sequence model uses the object semantic attention encoder followed by LSTM sequence language module [15]. IMFRE-Transformer [20] acquires aligned visual semantic features and subsequently leverages both global and local information to enrich the initial visual features and results in a more comprehensive visual feature representation. The image graph Transformer method [8] in the encoding phase utilizes graph representations to address the complex spatial connections among image regions. This approach involves integrating three sub-Transformer layers in parallel within each Transformer layer. The work in [23] introduces Semantic-Conditional Diffusion Networks (SCD-Net), a departure from traditional image captioning techniques that leverages semantically relevant sentences via cross-modal retrieval to guide the diffusion Transformer in generating captions to enhance the alignment between visuals and language in image captioning. The experimental results are shown in Table 1.

The outcomes of the model evaluation show that the proposed method not only aligns with but also surpasses the performance of nearly all the methods outlined in Table 1, as indicated by various evaluation metrics. Notably, our proposed semantic Transformer model attains the highest CIDEr score of 132.0, along with impressive scores of 28.9 and 58.5 on the METEOR and ROUGE-L metrics, respectively. The evaluation primarily focuses on METEOR, ROUGE-L, and CIDEr. As previously explained, METEOR and ROUGE-L rely on word sequences and synonym similarity in measuring the quality of generated captions. CIDEr, distinctively, transcends exact matches by incorporating semantic similarity between the generated and reference captions, ensuring a more comprehensive evaluation of caption quality. While the model in [8] achieves higher values by 0.2 and 0.5 in the METEOR and ROUGE-L metrics, respectively, our proposed model maintains statistical significance despite its simpler design. In contrast to [8], which incorporates three sub-Transformer layers in parallel to handle spatial relationships, our model offers a more streamlined approach.

#### 4.3.2. Ablation Studies

We explain the performance of a series of experiments aimed at quantitatively evaluating the accuracy of the generated captions in the proposed model using the MS-COCO dataset. Initially, we investigated the effect of semantic representations on captioning model performance. Then, we tested the influence of different CNN models on caption quality. Subsequently, we assessed the impact of fine-tuning on caption quality. Finally, we explored the effects of varying the number of Transformer multihead attention mechanisms on the generated captions, as described below:

**Effect of semantic representations on captioning model performance**. To evaluate the effect of adding semantic features to the encoder, we designed an encoder module without semantic representations, which contains attention mechanism layers based on image visual representation only. We preserved the experiment setting related to the number of layers, the number of attention heads, epochs, and Faster R-CNN backbone ResNet model. From Table 1, we observe that semantic representation provides positive effects, and adding an encoding module with semantic features improves the CIDEr score of “base” by 13.2. In the proposed architecture, the decoder is less likely to be misled by irrelevant attention results as the attention is supported by external knowledge base concepts in the semantic encoder module, increasing the relevant and connected information in the results.

**Evaluating the impact of CNN models on caption quality.** The size of Faster R-CNN and ResNet models depends on various factors, such as the depth of the network, number of layers, input image size, etc. Therefore, experiments using different ResNet models in image captioning are performed to evaluate the impact of the depth and complexity of the neural network on the quality of the generated captions. We selected pre-trained CNN ResNet models because they have deeper architectures with more layers, which allows them to capture more complex visual patterns in the region of interest. Additionally, ResNet models have been pre-trained on large-scale ImageNet datasets, containing a diverse range of object categories, which aids the models in learning a broad range of visual features useful for object detection and region-based feature extraction in image captioning tasks. In this experiment, we used three different types of Faster R-CNN backbone ResNet models, including ResNet-18, ResNet-50, and ResNet-101. It was hypothesized that using a larger CNN model would result in better caption generation. The summary of the experimental results is listed in Table 2, showing that ResNet-50 and ResNet-101 perform much better than ResNet-18, hence validating our original hypothesis.

**Figure 2:**
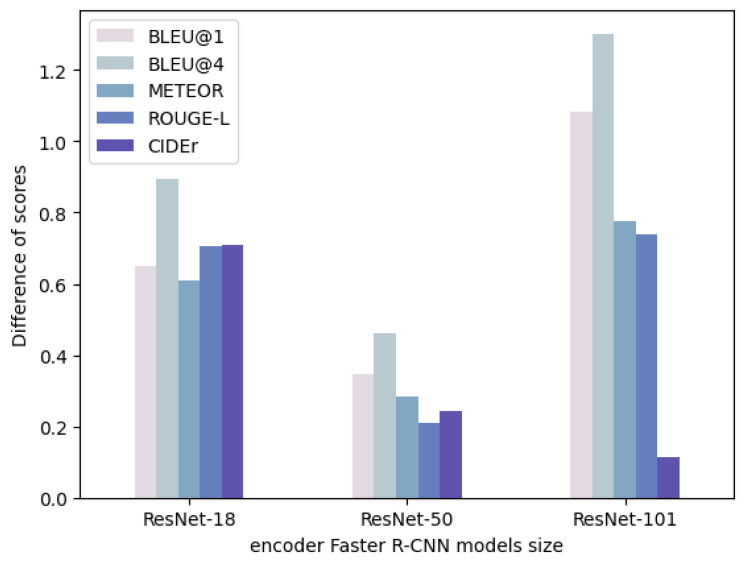
Effect of fine-tuning encoder CNN models on image captioning quality.

**Effect of fine-tuning on caption quality.** In this experiment, we aimed to investigate the impact of fine-tuning on the caption quality of the three ResNet encoders discussed earlier. We hypothesized that fine-tuning would improve the captioning quality since ResNet is trained on ImageNet and the MS-COCO dataset contains images that are not present in ImageNet. To evaluate the effect of fine-tuning, we compared the scores of the fine-tuned models for each encoder with the corresponding scores of the non-fine-tuned models. The results are summarized in Table 3 and Figure 2. They illustrate that fine-tuning is advantageous for all encoder types as it performs better than the baseline models. Notably, we observed that the deeper models benefit more from fine-tuning since it is challenging to transfer knowledge from a pre-trained model on large-scale ImageNet datasets to a task that involves different dataset content. The benefit can be clearly observed from the trend regarding BLEU@4 for all the encoder models.

**Effect of number of Transformer multiheads on caption quality.** In this experiment, we aimed to test the effect of varying the number of Transformer multihead attention units on the generated caption by the proposed method. We kept the encoder fixed as ResNet-101 and varied the decoder and tested the decoder with four, eight, and sixteen heads. Through varying the number of decoder Transformer heads, we can analyze the effects of language module on overall model performance, and optimize the architecture to achieve the best image captioning results. This is because the decoder component of an image captioning model is responsible for generating textual descriptions based on the visual features extracted from the images. We hypothesize that increasing the number of heads can potentially increase the expressive power of the model, enabling it to capture more complex relationships between visual and textual features. More heads can enable the model to capture more fine-grained details and dependencies, potentially leading to better image captioning results. The experimental results in Table 4 indicate that, for each encoder model, an increase in the number of heads leads to improved image captioning outcomes.

#### 4.3.3. Qualitative Evaluation

**Figure 3:**
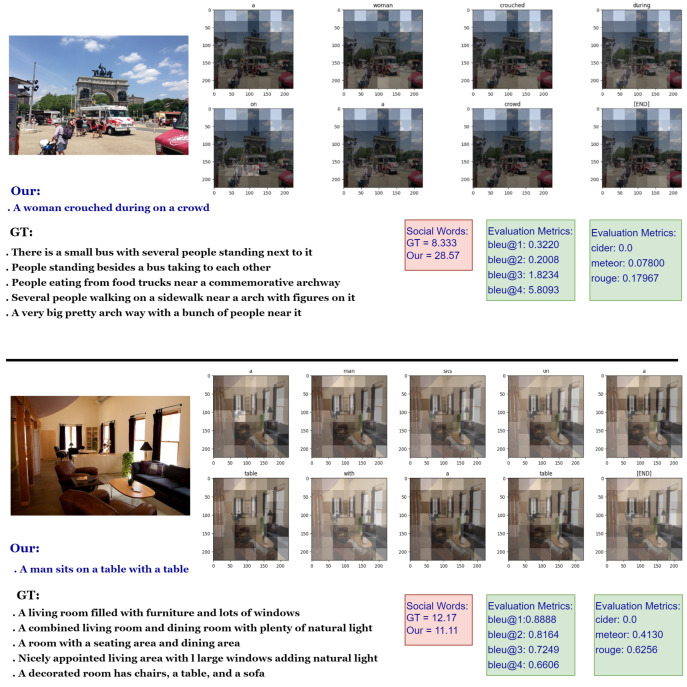
Examples of visualization results on the MS-COCO dataset are provided. Captions generated by our proposed model are displayed alongside manually annotated ground truth captions. The figure shows the evaluation metric scores for both sets of captions, as well as the percentage of social words that appear in each caption.

A qualitative analysis was conducted by examining sample images from the MS-COCO test set, together with their corresponding ground truth and generated captions. That provides a deeper understanding of the generated image captions and facilitates a direct comparison between the generated captions and ground truth descriptions. Figure 3 shows the analysis of the successful examples, indicating that incorporating visual semantic features can improve the model’s ability to handle complex image scenes with multiple objects and backgrounds, leading to more coherent and informative captions. Overall, the integration of visual semantic features can significantly enhance the performance of image captioning models and improve their ability to generate captions that accurately reflect the visual content of the image.

Furthermore, in this specific analysis, it is essential to evaluate both the quality of the generated captions and the ground truth captions based on their social meaning. Descriptions with a high frequency of social words often indicate that the text describes an event or situation in an approachable and socially engaging manner [55]. This analysis of social words encompasses language that involves interactions with others, such as pronouns (e.g., they, she, and us), verbs related to social engagement (e.g., talk and friends), and related terms. These are extracted by using the Linguistic Inquiry and Word Count (LIWC) dictionary [56]. Figure 3 illustrates how the LIWC dictionary reflects the percentage of social words present in both the ground truth and generated captions separately. The analysis of the indoor image example reveals that both the ground truth and the model-generated descriptions present a low frequency of social words, indicating a more formal and less socially oriented tone. These texts primarily focus on conveying informational content, with fewer elements related to social interactions. Oppositely, descriptions featuring a high frequency of social words tend to convey a more social or interactive tone. For instance, the description of a city crowd receives a higher social score than the ground truth, attributed to the classification of the word ‘crowd’ under the ‘social process’ category in the LIWC dictionary. This dictionary categorizes words into linguistic and psychological dimensions, providing valuable insights into the psychological and emotional tone of a text based on word usage. Therefore, conducting a qualitative linguistic analysis of the descriptions is crucial to gain a deeper understanding of the proposed approach.

The interpretable visualization analysis is also presented in Figure 3 to assess the effectiveness of using a semantic visual attention encoder for caption generation. The attention maps from the self-attention layers are displayed, with brighter areas indicating higher attention weights assigned to detected objects. In the first example, the model allocates more attention to the ‘woman’ and the ‘crowd’ while paying less attention to the ‘car’ in the image. In the second example, the attention is primarily directed towards the ‘table’ object, with less focus on the ‘windows’ and ‘sofa’. These observations suggest that the semantic visual attention encoder in the model adeptly captures the relevant visual content associated with the generated captions.

## 5. Discussion

In this work, we present a new image-Transformer-based model boosted with image object semantic representation. We extended the semantic Transformer model proposed by [15]. The core idea behind this proposed architecture is to enhance the attention mechanism of the original Transformer layers, specifically designed for image captioning. In the encoder, we augment the Transformer layer with semantic representations of image objects’ labels to capture the spatial relationships between these objects. For that, we conducted extensive experiments to demonstrate the superiority of our model, presenting both qualitative and quantitative analyses to validate the proposed encoder and decoder Transformer layers. When compared to previous top models in image captioning, our model achieves a high CIDEr score. This indicates that the proposed model can generate captions that are not only accurate but also diverse, coherent, and contextually relevant. This improvement is attributed to the utilization of external commonsense knowledge.

In the evaluation of the impact of different CNN models on caption quality, the experimental results demonstrate that captions generated by a ResNet-101 encoder consistently outperform those from ResNet-18 and ResNet-50 encoders in all tested scenarios. This validates our original hypothesis. The superior performance of the proposed method can be attributed to the residual connections in ResNet, enabling the creation of a deeper model. Additionally, the ResNet-101 CNN model excels in preserving significant visual information, resulting in better feature extraction for image captioning by learning more abstract and distinctive visual features. This is particularly advantageous for generating accurate and descriptive captions for complex images where identifying and describing subtle visual details is essential. However, the choice of a Faster R-CNN backbone for feature extraction depends on the specific task and available resources. More complex backbones like ResNet-101 or ResNet-50 may yield better performance but may also require additional computational resources and longer training times. In addition, the fine-tuning visual features using CNNs like ResNet improve the relevance and quality of the generated captions, as evidenced by higher BLEU@4 metric scores across various encoder models.

Furthermore, we have observed that increasing the number of Transformer heads in the model enhances accuracy across various evaluation metrics. However, it comes at the cost of increased training time. Each additional head introduces extra parameters that require optimization during training, thus extending the training process. Furthermore, during the inference phase, generating captions with models featuring a high number of attention heads can result in slower performance, which may pose a notable drawback in real-time applications.

In summary, integrating visual semantic features significantly enhances the performance of Transformer-based image captioning models, enabling them to generate captions that faithfully represent the visual content of the images.

## 6. Generalization

To demonstrate the broad applicability of the proposed semantic Transformer model, we conducted experiments on the MACE dataset [25]. This dataset comprises images from a visual historical archive that are not included in ImageNet. It is generated from archival video data.

In particular, generating content captions for heuristic data is an open problem with various challenges: (i) the lack of truly large-scale datasets; (ii) some old video content sounds/scenes are not clear or become damaged when converted and run via new-technology devices; and (iii) the data have outdated objects and scenes and also include cultural and historical context.

In Table 5, one can observe the evaluation results for captions generated by ResNet-101, ResNet-50, and ResNet-18, which were used to extract feature vectors from frames in each video. These vectors were then passed through encoder semantic Transformer and decoder Transformer modules.

The results from ResNet-50 are higher than ResNet-18 and ResNet-101 in most evaluation metrics. The reason regarding MACE data is that they comprise a small dataset that might not provide enough diverse examples to leverage the additional capacity of ResNet-101. Additionally, the deeper and more complex nature of ResNet-101 in a small-dataset context raises the risk of overfitting, potentially capturing noise instead of generalizing well to unseen data.

## 7. Conclusions and Future Work

In this work, we introduce a new Transformer-based model for image captioning. Our approach incorporates semantic representations of image objects to capture spatial relationships between objects, aiming to enhance attention mechanisms for image captioning. Extensive experiments on the MS-COCO dataset confirm that the proposed model achieves an impressive CIDEr score of 132.0, indicating that it generates accurate, diverse, coherent, and contextually relevant captions through the use of external commonsense knowledge. A ResNet-101 encoder consistently outperforms ResNet-18 and ResNet-50 encoders in caption quality, attributed to its residual connections and better feature extraction. Additionally, refining with ResNet enhances BLEU@4 metric scores, thereby enhancing caption quality. Moreover, augmenting the number of Transformer multihead attention mechanisms improves image captioning outcomes. Nevertheless, this heightened accuracy is accompanied by the cost of extended training time, which can negatively affect real-time applications.

The study also applies the model on the MACE dataset to generate descriptive sentences for video frames, improving accessibility and understanding of historical artifacts through experiments. In summary, integrating visual semantic features enhances image captioning model performance, and provides reliable representations of visual content.

Future work will also examine the use of new models that have been successfully applied to different applications. These include (a) the PF-BiGRU-TSAM model, which has been used for interactive remaining useful life prediction of lithium-ion batteries [57]; this model uses data-driven deep learning methods and time windows for prediction tasks over time; (b) the neural network in lifetime extension approach, based on Leven–Marq neural network and power routing [58]; this model uses the Levenberg–Marquardt algorithm for optimizing the backpropagation neural network for real-time prediction in an industrial system.

## Figures and Tables

**Table 1:** The performance of proposed model and other methods on MS-COCO. All values are reported as a percentage.

Method	BLEU@1	BLEU@4	METEOR	ROUGE-L	CIDEr
Base	79.4	35.8	28.0	58.0	118.8
Semantic Transformer [15]	78.6	36.0	27.6	57.7	120.9
IMFRE-Transformer [20]	77.1	36.4	28.3	57.1	117.1
Image Transformer [8]	80.8	39.5	29.1	59.0	130.8
SCD-Net [23]	79.0	37.3	28.1	58.0	118.0
Ours	80.0	37.7	28.9	58.5	132.0

**Table 2:** Experimental results for varying encoder in Faster R-CNN object detector model size on MS-COCO test split. All values are reported as a percentage.

Encoder	BLEU@1	BLEU@2	BLEU@3	BLEU@4	METEOR	ROUGE-L	CIDEr
ResNet-18	74.88	57.17	54.30	29.85	21.10	47.29	111.70
ResNet-50	77.97	59.20	57.74	34.63	24.83	50.10	121.22
ResNet-101	80.04	60.29	60.01	37.70	28.99	58.50	132.04

**Table 3:** Experimental results for fine-tuning encoder in Faster R-CNN object detector model size on MS-COCO test split. It shows the difference in scores between the baseline model scores (Table 2) and fine-tuned scores. All values are reported as a percentage.

Encoder	△BLEU@1	△BLEU@2	△BLEU@3	△BLEU@4	△METEOR	△ROUGE-L	△CIDEr
ResNet-18	0.650	0.760	0.836	0.894	0.611	0.707	0.711
ResNet-50	0.346	0.397	0.429	0.461	0.283	0.208	0.244
ResNet-101	1.081	1.198	1.136	1.302	0.774	0.740	0.114

**Table 4:** Experimental results for the proposed model for varying number of multihead attention units in decoder Transformer for MS-COCO test data for the proposed architecture. All values are reported as a percentage.

Encoder	Head Number	BLEU@1	BLEU@2	BLEU@3	BLEU@4	METEOR	ROUGE-L	CIDEr
ResNet-18	4	74.32	60.99	59.80	26.59	18.89	41.00	98.20
	8	74.88	57.17	54.30	29.85	21.10	47.29	111.70
	16	76.14	63.09	55.36	29.93	23.30	47.18	122.01
ResNet-50	4	75.29	59.44	58.00	30.91	21.28	59.02	107.90
	8	77.97	59.20	57.74	34.63	24.83	50.10	121.22
	16	82.10	61.04	58.37	34.13	26.85	54.12	123.86
ResNet-101	4	75.14	62.04	61.00	28.73	25.11	56.39	115.60
	8 *	80.04	60.29	60.01	37.70	28.99	58.50	132.04
	16	79.13	71.94	71.03	42.97	31.00	60.75	134.82

* The baseline architecture.

**Table 5:** Experimental results for different encoder Faster R-CNN object detector models’ size on MACE frame–text pair test split. All values are reported as a percentage.

Encoder	BLEU@1	BLEU@2	BLEU@3	BLEU@4	METEOR	ROUGE-L	CIDEr
ResNet-18	74.38	71.54	69.73	68.51	46.19	71.89	62.24
ResNet-50	81.30	79.40	78.17	77.24	53.21	79.42	71.16
ResNet-101	75.50	72.74	71.10	69.93	49.81	76.95	76.01

## Data Availability

The dataset MS-COCO [24] is openly available in a public repository and it can be downloaded at https://www.cocodataset.org/download. The dataset MACE [25] is private and it can be provided by request to the authors, with the permission of the MACE—Media Archive for Central England www.macearchive.org.
